# A New Green Model for the Bioremediation and Resource Utilization of Livestock Wastewater

**DOI:** 10.3390/ijerph18168634

**Published:** 2021-08-16

**Authors:** Linhe Sun, Huijun Zhao, Jixiang Liu, Bei Li, Yajun Chang, Dongrui Yao

**Affiliations:** 1 Jiangsu Key Laboratory for the Research and Utilization of Plant Resources, Institute of Botany, Jiangsu Province and Chinese Academy of Sciences (Nanjing Botanical Garden Mem. Sun Yat-Sen), Nanjing 210014, China; linhesun@cnbg.net (L.S.); zhaohj0707@163.com (H.Z.); ljx891654338@163.com (J.L.); libei@cnbg.net (B.L.); 2 Jiangsu Engineering Research Center of Aquatic Plant Resources and Water Environment Remediation, Nanjing 210014, China; 3 College of Geography and Environmental Science, Northwest Normal University, Lanzhou 730070, China

**Keywords:** *Oenanthe javanica*, livestock wastewater, phytoremediation, resource utilization

## Abstract

The rapid growth of the livestock and poultry industries has resulted in the production of a large amount of wastewater, and the treatment of this wastewater requires sustainable and environmentally friendly approaches such as phytoremediation. A substrate-free floating wetland planted with water dropwort (*Oenanthe javanica*), a common vegetable in Southeast China, was constructed to purify a lagoon with anaerobically and aerobically treated swine wastewater in Suqian, China. The average removal rates of total nitrogen, ammonium nitrogen, nitrite nitrogen, and chemical oxygen demand were 79.96%, 95.04%, 86.14%, and 59.91%, respectively, after 40 days of treatment. A total of 98.18 g∙m^−2^ nitrogen and 19.84 g∙m^−2^ phosphorus were absorbed into plants per harvest through the rapid growth of water dropwort biomass, and the nitrogen accumulation ability was similar to that observed of other plants, such as water hyacinth. In addition, the edible part of water dropwort was shown to comply with the Chinese National Food Sanitation Standards and be safe for human consumption. Its low soluble sugar content also makes it a suitable addition to the daily diet. Overall, substrate-free floating constructed wetlands planted with water dropwort could be more widely used for livestock wastewater purification and could be integrated with plant–livestock production in China because of its high removal efficiency and recycling utilization of water dropwort biomass.

## 1. Introduction

China has been the world’s largest livestock and poultry producer since the early 1990s, and livestock and poultry production has continually increased given the rapidly growing demand for animal products [1,2]. The environmental pollution associated with the rapid development of livestock and poultry-breeding farms has become increasingly severe in China, and this greatly impedes the sustainable development of the livestock and poultry breeding industry. Livestock wastewater (LW) contributes approximately 41.9%, 21.7%, and 37.9% of the chemical oxygen demand (COD), total nitrogen (TN), and total phosphorus (TP) discharged from all types of wastewater, respectively [3]. With its large discharge and high pollution loads, LW has become the main source of non-point source pollution in China [4]. Although physico-chemical methods, such as filtration, adsorption, electrodialysis, and photocatalysis, are widely used to treat LW because of their high efficiency, these methods are not environmentally friendly and may cause secondary pollution [3]. There is, thus, a pressing need to develop bioremediation approaches for LW that are eco-friendly and low-cost, given that physico-chemical methods cannot be feasibly used to treat LW across the large numbers of small-scale and family-operated farms in China [5,6,7].

Phytoremediation is a technology that involves using living plants to eliminate contaminants in soil, air, and water, and it has been increasingly used for LW purification for its lower costs, being more environmentally friendly compared with physico-chemical methods, energy-saving properties, and aesthetic value [8,9,10]. The selection of plants for phytoremediation requires several considerations. First, the tolerance and sensitivity of different plants to contaminants can vary. Second, an increasing pollutant removal efficiency requires plants with fast growth rates, a high biomass, an extensive rhizosphere, and easy management and harvest [3,11]. Third, native species are often preferred for bioremediation [3,12] because non-native or exotic species hinder the restoration of many natural areas and may cause irreversible ecological damage [13]. Fourth, the seasonal withering and falling of plants not only cause secondary pollution but also disrupt the efficacy of phytoremediation [6,14]. In addition, plants used for remediation engineering need to be harvested in a timely manner and recycled economically to address the problems relating to resource utilization and biomass disposal [3]. Thus, the selection of plants is critically important for improving the efficacy of phytoremediation [15]. Over the past two decades, the effectiveness of various aquatic plant species for LW phytoremediation has been investigated. Many of these plants have been shown to have a high efficiency in removing contaminants through laboratory tests; however, tests of the efficacy of these plants in removing contaminants in livestock farms are lacking [16,17,18,19].

Water dropwort (*Oenanthe javanica* (Blume) DC) is an aquatic plant cultivated in East Asian countries. It is a common edible vegetable with a high nutritional and medicinal value in China [20,21,22]. Aside from its economic value, its ability to grow rapidly in polluted water, its flourishing roots, high nitrogen (N) removal efficiency, tolerance to freezing temperatures during winter, and capacity for repeated harvest make water dropwort an ideal plant for wastewater phytoremediation in China [23,24]. However, few studies have assessed the potential for water dropwort to be used for LW purification; in situ experiments in LW lagoons of livestock and poultry farms are especially rare.

Here, in situ experiments using a substrate-free floating constructed wetland planted with water dropwort were performed to evaluate the feasibility of LW purification using water dropwort. LW purification efficiency and the plant biomass production of the wetland were determined. The content of various nutrients and the food safety of water dropwort following LW treatment were also estimated. The results of this study will help improve the use of phytoremediation technologies for sustainable LW treatment in China.

## 2. Materials and Methods

### 2.1. Experimental Design and Set-Up

The LW used in this study was anaerobically and aerobically treated swine wastewater obtained from a swine farm in Suqian City, Jiangsu Province, China, and poured into a square 100 m^2^ lagoon 80 cm in depth that was seepage-proofed using an HDPE membrane situated around and at the bottom of the lagoon. Suqian is located in the northern part of Jiangsu Province (33°12′~34°25′ N, 117°6′~119°13′ E) and experiences a temperate monsoon climate zone. The average temperature is 14.2 °C, and the average annual precipitation is 910 mm. Before this study, the lagoon was used for swine manure storage and anaerobic digestion. The content of TN, ammonium nitrogen (NH_4_^+^-N), nitrite nitrogen (NO_2_^−^-N), TP, and COD in the LW before treatment was 78.53 ± 1.49 mg∙L^−1^, 25.20 ± 0.14 mg∙L^−1^, 1.57 ± 0.28 mg∙L^−1^,247.78 ± 7.59 mg∙L^−1^, and 191.11 ± 5.33 mg∙L^−1^, respectively.

### 2.2. Construction of the Integrated Floating Wetland System

**Figure 1:**
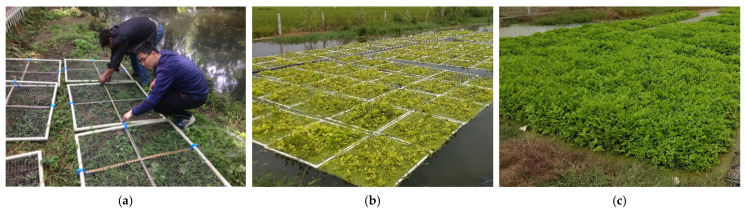
Construction of the integrated floating wetland system. (**a**) The 1 m × 1 m square floating beds units preparation; (**b**) experiment site on day 0, plants were pre-cultured for one week; (**c**) experiment site on day 35.

The floating beds consisted of 1 m × 1 m square units, and the wetland system consisted of 100 units of floating beds. The frames were composed of PVC pipes with a diameter of 4 cm, and the beds inside were nylon nets with 1.5~2.0 cm mesh. All floating beds units were fastened by crossing bamboo poles (Figure 1a).

The evergreen water dropwort material was a local cultivar in Suqian. Its suitable growth temperatures are 5~30 °C, and it can survive temperatures of −15~40 °C. Forty strong stems 18~22 cm in length were planted as propagules on each floating bed. Stem propagules were planted horizontally and remained inside the floating beds. The total original biomass of the stem propagules on each floating beds unit was about 5 kg. Plants were pre-cultured for adaptation before experimentation. After one week (on 11 September 2019), the planted floating beds were moved into the LW lagoon for 40 days of treatment (Figure 1b,c).

### 2.3. Sampling and Determination Methods

Water samples were collected 30 cm below the water surface in 3 different positions of the lagoon every 10 days at 10:00 am; the samples were then stored at 4 °C and analyzed within 48 h. TN, NH_4_^+^-N, NO_2_^−^-N, TP, and COD were analyzed to estimate the pollutant removal of water dropwort planted in the substrate-free floating constructed wetland. TN was determined using alkaline potassium persulfate digestion UV spectrophotometry (HJ636-2012), TP was determined using ammonium molybdate spectrophotometry (GB11893-89), and COD was determined using the acidic potassium permanganate method [25,26]. Concentrations of NH_4_^+^-N and NO_2_^−^-N were measured by Naismith spectrophotometry with medium-range (HI96715) and low-range portable photometers (HI96707) (HANNA Instruments, Woonsocket, RI, USA).

Water dropwort plants were harvested 40 days after they were planted on the floating beds. Fresh plant samples were weighed, dried at 65 °C, and re-weighed to determine the moisture content. Total organic carbon (TOC) was determined using the volumetric method with potassium dichromate, the N content was determined using the Kjeldahl method after digestion with H_2_SO_4_-K_2_SO_4_-CuSO_4_-Se [27,28], the phosphorus (P) content was determined using ammonium molybdate spectrophotometry after digestion with H_2_O_2_-H_2_SO_4_ [29], the concentrations of heavy metals were determined using flame atomic absorption spectrometry [30], and antibiotic and nutrient compositions were quantified by high-performance liquid chromatography–tandem mass spectrometry (HPLC-TMS) [31]. Edible tissues were sent to Center Testing International Pinbiao (Jiangsu) Certification Technology Co., Ltd., to test for pesticide residues.

### 2.4. Health Risk of Antibiotic Assessment

The potential level of human exposure to antibiotics through the consumption of water dropwort was determined following the method of Pan et al. [31]. The level of human exposure was calculated as

human exposure = *C* × *D* × *T* (1)

where *C* represents the content of antibiotics in the edible tissue (ng∙g^−1^ wet weight), *D* is the average daily consumption of water dropwort (g∙day^−1^ wet weight), and *T* is the exposure time (day). The average daily vegetable intake of adults was assumed to be 389 g∙day^−1^ (FW), and the average adult body weight was assumed to be 55.9 kg [31,32].

### 2.5. Data Analysis

Statistical analysis was performed using Microsoft Excel (Microsoft Corporation, Redmond, DC, USA) and SPSS 26.0 software (SPSS Inc., Chicago, IL, USA). One-way analysis of variance (ANOVA) and Duncan’s test were performed to determine statistically significant differences (*p* < 0.05). All figures were constructed by Origin 9.0 (OriginLab Corporation, Northampton, MA, USA).

## 3. Results

### 3.1. Purification Performance of the Substrate-Free Floating Constructed Wetland

**Figure 2:**
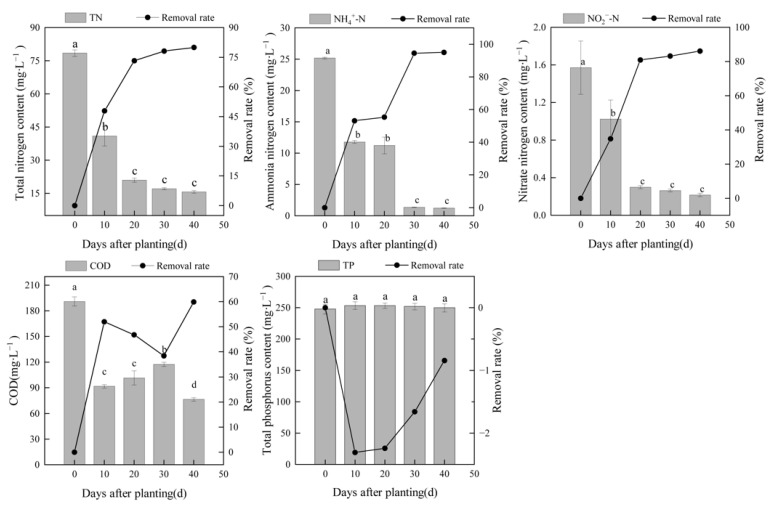
Concentrations and removal rates of TN, NH_4_^+^-N, NO_2_^−^-N, TP, and COD in the LW lagoon treated by a substrate-free floating constructed wetland planted with water dropwort. Different letters indicate significant differences between different days after planting (*p* < 0.05).

To investigate the purification performance of the substrate-free floating constructed wetland, the concentrations of nutrients and COD in LW were determined every 10 days (Figure 2). The average concentration of COD, which is an indicator of organic pollution in water, decreased from 191.11 mg∙L^−1^ before treatment to 76.61 mg∙L^−1^ after treatment, which met the level II discharge standard of pollutants for municipal wastewater treatment plants in China (100 mg∙L^−1^, GB 18918-2002). The COD concentration decreased rapidly in the first 10 days after planting the water dropwort; the removal rate after 10 days was 51.95%, and the removal rate after 40 days was 59.91%, suggesting that the wetlands with water dropwort eliminated organic pollutants in LW. The TN concentration of LW decreased from 78.53 mg∙L^−1^ before treatment to 15.74 mg∙L^−1^ after 40 days of treatment with a removal rate of 79.96%. The NH_4_^+^-N concentration decreased from 25.20 mg∙L^−1^ to 1.25 mg∙L^−1^ with an average removal rate of 95.04%. The NO_2_^−^-N concentration decreased from 1.57 mg∙L^−1^ to 0.22 mg∙L^−1^ with an average removal rate of 86.14%. There were no significant differences in the TN concentrations between day 20 and day 40, suggesting that the water dropwort floating wetland could remove the TN efficiently in 20 days. The NH_4_^+^-N concentration became stable after 30 days of treatment, which was 10 days later compared with the TN and NO_2_^−^-N concentrations. The main reason may be that the wetland covered more than 90% of the water surface, which suppressed the evaporation of NH_3_; nitrification was inhibited because of the low dissolved oxygen in the LW.

There were no significant differences in TP in the lagoon before and after treatment (*p* > 0.05; Figure 2). The biomass of the floating constructed wetland was high because of the rapid growth of water dropwort, and the plants needed to uptake P from the LW during the experiment. However, the TP concentration changed from 247.78 mg∙L^−1^ before treatment to 249.87 mg∙L^−1^ after 40 days of treatment, suggesting that additional P was released in the lagoon. As no other external P was added to the lagoon, internal P from the sediments might maintain the high TP concentrations in the wetland system. According to the owner of the farm, the lagoon had been used for swine manure storage and anaerobic fermentation for 1 year prior to our study, which led to the accumulation of sediments rich in P.

### 3.2. Biomass and Nutrient Storage of Water Dropwort in the Constructed Wetland

The biomass and content of nutrients in different organs of water dropwort were determined 40 days after planting to characterize the nutrient storage ability of the substrate-free floating constructed wetland planted with water dropwort (Table 1). The fresh matter (FM) of the root, stem, and leaf tissue of water dropwort was 13.55 ± 1.22 kg∙m^−2^, 10.10 ± 0.43 kg∙m^−2^, and 5.95 ± 0.34 kg∙m^−2^, respectively; the dry matter (DM) of the root, stem, and leaf tissue was 1.05 ± 0.03 kg∙m^−2^, 0.50 ± 0.02 kg∙m^−2^, and 0.61 ± 0.03 kg∙m^−2^, respectively. The TN content of water dropwort in the root, stem, and leaf tissue was 45.19 ± 0.31 g∙kg^−1^ DM, 35.87 ± 0.41 g∙kg^−1^ DM, and 53.76 ± 0.20 g∙kg^−1^ DM, respectively. It was similar to the values observed for the N content in water hyacinth (*Eichhornia crassipes* (Mart.) Solms), which has been widely applied for LW purification in the last 15 years in China [33,34,35]. The P content was 8.73 ± 0.14 g∙kg^−1^ DM in root tissue, 10.01 ± 0.25 g∙kg^−1^ DM in stem tissue, and 9.30 ± 0.08 g∙kg^−1^ DM in leaf tissue, indicating that 1.98 kg P was absorbed by water dropwort from the LW. In addition, the average C (542.46 g·kg^−1^), N (41.35 g·kg^−1^), and P (7.03 g·kg^−1^) content was 1.5, 1.6, and 2.1 times higher in the water dropwort grown in LW than in the aquatic plants in inland waters of China, respectively [36]. Additional results indicated that the total N and P storage of the water dropwort was 98.18 g·m^2^ and 19.84 g·m^2^, which was similar to values observed for water hyacinth (Table 2). Although the concentration of TP in the lagoons did not change significantly during the experiment, there was noticeable P removal through the plant uptake pathway (1.98 kg). This indicates that the amount of endogenous P released from the bottom of the pond was far more than that absorbed by water dropwort. These results suggest that water dropwort is highly tolerant of high nutrient conditions and is an excellent green material for the bioremediation of LW.

### 3.3. Safety Assessment and Resource Utilization of Water Dropwort

In addition to large amounts of carbon, N, and P, LW also contains a large amount of mineral nutrients and heavy metals (e.g., Cd, As, Cu, Pb, Cr) derived from aquaculture feed additives [37,38,39]. However, water dropwort is a medicinal and edible plant widely distributed in China, especially in Southeast China. When water dropwort grown on LW in a substrate-free floating constructed wetland is used as vegetable or green fodder, the heavy metals may enter the human body through the food chain, endangering human health. Thus, the content of the heavy metals Cd, As, Cu, Pb, and Cr in water dropwort was quantified in this study (Table 3). The content of these heavy metals was significantly higher in root tissue than in stem and leaf tissue. As the tissue that makes direct contact with and absorbs nutrients from LW, root tissue accumulated significantly more heavy metals than both stem and leaf tissue. The Cu, Cd, Cr, and As content was significantly lower in stem tissue than in root and leaf tissue, and there were no significant differences in the Pb content between stem and leaf tissue. Given that the stem is the main part of water dropwort that is consumed in China, the content of these four heavy metals (Cu, Cd, Cr, and As) in the stem was measured. The Cu, Cd, Cr, and As content was 0.032 ± 0.006 mg∙kg^−1^, 0.054 ± 0.003 mg∙kg^−1^, 0.102 ± 0.002 mg∙kg^−1^, and 0.015 ± 0.001 mg∙kg^−1^, respectively, which were below the limits of the Chinese National Food Sanitation Standards for Heavy Metals, indicating that the health risks through ingestion are low.

In 2013, more than half of the antibiotics produced in China were used for animals [40]. Although animal antibiotics consumption has decreased rapidly since 2014, the total antibiotic use in animals is still high [41]. The antibiotics have the potential to be taken up by dropwort planted on LW. To estimate the potential human exposure to antibiotics through the consumption of water dropwort, the content of 16 antibiotics was determined (Table 4); 11 antibiotics could not be detected in both the aboveground tissues and underground tissues. The oxytetracycline, doxycycline, and tetracycline content was significantly lower in the aboveground parts than in the underground parts (*p* < 0.05). As aboveground tissues are edible, we used the content of these antibiotics in the aboveground tissues to calculate potential human exposure. Daily human exposures of sulfadimidine, oxytetracycline, doxycycline, tetracycline, and aureomycin were 0.4928 μg, 0.1582 μg, 0.4624 μg, 0.3799 μg, and 0.4603 μg, respectively, which were much lower than the acceptable daily intake (ADI) levels established by the United Nations Food and Agriculture Organization [42].

Although no pesticides were used during the growth of water dropwort, the concentrations of 68 different pesticides in water dropwort were determined by a third-party testing agency to meet China’s food safety standards for vegetable products. The results showed that the content of all 68 pesticide residues was below the detection threshold (Table S1). The nutritional composition of water dropwort was also examined (Table 5). The stem contained more carbohydrates, vitamin C (VC), and crystalline cellulose than root and leaf tissues. However, compared with other common stem vegetables in China, water dropwort contains fewer carbohydrates [43]. Given that the Dietary Guidelines for Chinese Residents recommend limiting sugar intake, the low soluble sugar content of water dropwort makes it a suitable addition to the daily diet [44]. Although small amounts of heavy metals and antibiotics from the LW can be absorbed into the edible part of water dropwort, the content of these contaminants met safety standards for human consumption.

**Figure 3:**
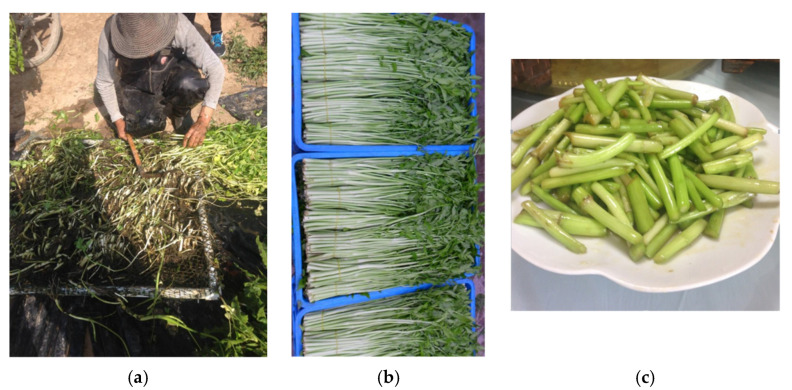
Harvest process of water dropwort for LW purification. (**a**) Reaping the plants from the floating beds; (**b**) water dropwort vegetable products for sale; (**c**) A common dish procured from water dropwort in China.

The yield of water dropwort was 16.05 kg∙m^−2^ on average containing both leaves and stems on each floating beds unit per-harvest. Only the fresh and soft part of water dropwort could be launched to the market as vegetable products (Figure 3). After discarding the withered and strong part, about 6 kg vegetable products could be collected. The discarded tissues could be used as animal feeds or propagules. The water dropwort can be harvested four times a year; thus, 1 m^2^ floating bed could output about 24 kg vegetable products per year. A 100 m^2^ lagoon can bring about 10,800 RMB income per year according to the water dropwort price in Jiangsu, 2020 (4.5 RMB∙kg^−1^).

## 4. Discussion

LW is high in COD and NH_4_^+^-N. Plants with a tolerance to special environment and biological characteristics are potentially suitable for LW management. Much research in the field of agriculture and environmental protection fields has focused on identifying plants with a strong nutrient purification ability that can be conveniently harvested and recycled for the treatment of LW as well as overcoming the problem of “ammonia toxicity” and the seasonal restrictions of plants. *Spirodela polyrhiza* can remove 89.4% and 83.7% of TP and TN from a 6% swine wastewater lagoon in 8 weeks, respectively, but the plants need to be harvested twice a week [45]. *Lemna* sp. showed a TN removal of 67.03% and TP removal of 43.62% from 10% swine wastewater in 21 days. However, *Lemna* sp. plants could only be cultured for 13 days, after which the biomass died and sank to the bottom of the experimental units, leading to secondary pollution [46]. *Lemna aequinoctialis* removed NH_4_^+^-N and TP efficiently from synthetic anaerobically digested swine wastewater with removal rates of 84% and 98% in 66 days, respectively; however, heavy metals (Zn^2+^) and antibiotics (oxytetracycline, OTC) inhibited their removal efficiency [18,47]. Water hyacinth and *Myriophyllum aquaticum* are widely used for LW phytoremediation because of their high drought tolerance and their rapid growth and reproduction. Lu et al. showed that an artificial water hyacinth wetland removed 21.78% of TN, 23.02% of TP, and 64.44% of COD from duck wastewater in 40 days [26]. In a simulated experiment, water hyacinth removed 63.74% of TN and 19.05% of TP from 10% swine wastewater in 21 days [46]. *Myriophyllum aquaticum* removed more than 97% of NH_4_^+^-N and 94% of TN from swine wastewater in 28 days [45]. However, water hyacinth and *Myriophyllum aquaticum* are invasive plants in China that lead to the local extinction of macrophytes and decrease the native aquatic macrophyte diversity [48,49]. Some ornamental plants have also been utilized for LW treatment. Both the mining ecotype and non-mining ecotype of *Polygonum hydropiper* could remove over 70% of TN and over 65% of TP from mixed LW collected from a wastewater treatment plant in 45 days [50]. Phytoremediation treatment with *Canna indica* could remove 62.84% of COD, 51.65% of NO_2_^−^-N, and 54.72% of TP from dairy industry wastewater after electrocoagulation treatment in 6 weeks. However, *Canna indica* started drying after 4 weeks, and the removal of old stems was required during the phytoremediation process [51]. Although ornamental plants are aesthetically pleasing, the utilization of their plant biomass resources still requires further study [3]. The use of the aforementioned plants for LW purification also faces problems associated with seasonal litter, harvest management, and resource utilization.

Water dropwort, a traditional Chinese vegetable, showed high efficiency in removing N from LW over 40 days, which corresponds to one growth cycle of water dropwort. The average content of N (41.35 g·kg^−1^) in water dropwort grown in LW was 1.6 times that of aquatic plants in inland waters of China [36], which reflects its efficient absorption and storage capacity of N. The ability of microbes in LW to reduce N through denitrification contributed to the elimination of N in the wetland. The extensive rhizosphere indicated by the high biomass of below-water tissues might harbor a high diversity of bacteria via the high root surface area available for microbes and the exudates provided by the rhizosphere. Thus, the emission of N_2_, NO_x_, and NH_3_ during nitrification–denitrification and ammonia volatilization stemming from crosstalk between water dropwort roots and rhizosphere microbes is likely an important contributor to N removal in wetland systems [6,52,53,54,55]. Although the concentration of TP in the lagoons did not change significantly during the experiment, the content of P was 2.1 times higher in water dropwort grown on LW than in aquatic plants in inland waters of China [36], and a noticeable P removal through the plant uptake pathway was observed (1.98 kg). As there were no sediments or substrates in the floating bed, the P absorbed and accumulated into the water dropwort was derived from sediments below the waterbody, suggesting that the sediments below released approximately 2 kg of P into the overlying LW over 40 days. There are four pathways for the removal of P: adsorption to the sediment or substrate, PH_3_ volatilization, physical and chemical precipitation, and plant uptake [56]. Plant uptake may have played a major role in P removal in this study, as the sediments did not adsorb P from the LW, but instead released it. Duan et al. showed that plant uptake by water dropwort was responsible for 68.81% of TP removal from polluted river water, which was much more than that of sedimentation (23.70%) [57]. Thus, the water dropwort-constructed wetland in this study had a strong tolerance to high nutrient conditions and a high P efficiency, although the TP concentrations of the LW remained stable. These results indicated that the water dropwort floating wetland had a strong absorption and purification capacity for both N and P in LW and is an ideal green material for the remediation of LW. Additional research on substrate-free floating constructed wetlands planted with water dropwort is needed to clarify the role of microbes in LW purification.

The utilization of aquatic plants for LW management is critically important for maintaining the stability of LW treatment, preventing secondary pollution, and maintaining ecological balance [3]. Aquatic plants used for LW purification were initially considered great resources for livestock feed because of their high content of proteins, starches, and celluloses. The yield and quality of eggs produced by ducks increased when they were fed water hyacinth that had been used for LW phytoremediation [26]. Biofuel is another product that can be produced by aquatic plants [58]. The use of duckweed, which has high levels of starch and cellulose, for swine wastewater treatment is more effective for increasing the ethanol yield than maize [59,60]. Biofuel is considered the most promising alternative to fossil fuels for its ability to be recycled and its environmentally friendly features; however, the development of advanced biorefining techniques for the isolation of biofuel precursors in a cost-effective manner is still a major challenge impeding the wide use of plant-based biofuels [61,62]. Dissimilar to other aquatic plants, such as duckweed and water hyacinth, water dropwort can be directly utilized because it is a common vegetable in southeast China, which means that it has a much higher biomass utilization efficiency after harvest. The stem of water dropwort is the main part consumed by humans; the remaining parts can be used as livestock feed. The yield and food safety of water dropwort planted on LW are key factors for evaluating its utilization efficiency. The Cd, As, Cu, Pb, and Cr content were quantified in LW (Table 4). The content of the heavy metals Cu, Cd, Cr, and As in the stem was 0.032 ± 0.006 mg∙kg^−1^, 0.054 ± 0.003 mg∙kg^−1^, 0.102 ± 0.002 mg∙kg^−1^, and 0.015 ± 0.001 mg∙kg^−1^, respectively, which were all below the limits of the Chinese National Food Sanitation Standards for Heavy Metals, indicating that health risks through the ingestion of water dropwort are low. We also found that the daily human exposure to antibiotics was much lower than the ADI levels established by the United Nations Food and Agriculture Organization if water dropwort is consumed daily [42]. A total of 68 pesticide residues in China’s food safety standards for vegetable products could not be detected. These findings, combined with the analysis of the nutrient composition, suggest that water dropwort on substrate-free floating wetland can not only purify the LW, but can also save existing farmland resources and provide safe and low-calorie vegetable products.

## 5. Conclusions

A substrate-free floating constructed wetland planted with water dropwort could efficiently remove N (79.96%) and COD (59.91%) from LW. The average C (542.46 g·kg^−1^), N (41.35 g·kg^−1^), and P (7.03 g·kg^−1^) content was 1.5, 1.6, and 2.1 times higher in water dropwort grown in LW than in aquatic plants in inland waters of China, respectively. The total N and P storage of the water dropwort was 98.18 g·m^2^ and 19.84 g·m^2^, respectively, which was similar to the values reported for water hyacinth. The floating wetland with water dropwort could not only efficiently absorb and purify N and P from LW, but could also produce a low-calorie vegetable that meets Chinese food safety standards. This research provides a new green model for the bioremediation and recycling of LW that could be applied more widely in China.

## Figures and Tables

**Table 1:** Content of C, N, and P in various organs of water dropwort at different growth stages.

Organ	C Content (g·kg^−1^)		N Content (g·kg^−1^)		P Content (g·kg^−1^)	
	Day 0	Day 40	Day 0	Day 40	Day 0	Day 40
Root	649.35 ± 30.02	425.79 ± 6.82 *	35.41 ± 0.75	45.19 ± 0.31 *	3.44 ± 0.33	8.73 ± 0.14 *
Stem	611.38 ± 31.24	392.11 ± 29.07 *	32.95 ± 0.35	35.87 ± 0.41 *	5.71 ± 1.53	10.01 ± 0.25 *
Leaf	622.01 ± 30.16	519.75 ± 27.79 *	48.68 ± 0.21	53.76 ± 0.20 *	7.64 ± 0.06	9.30 ± 0.08 *

* *p* < 0.05 compared with the day 0 by Student’s *t*-test.

**Table 2:** Comparison of N and P accumulation of water dropwort and water hyacinth.

Species	Accumulated Biomass (kg·m^−2^)		N Accumulation (g·m^−2^)	P Accumulation (g·m^−2^)	Reference
	Aboveground Part	Underground Part			
Water dropwort	16.05	13.55	98.18	19.84	This study
Water hyacinth	7.08~46.95	5.79~38.42	11.04~144.90	0.93~36.10	[33,34,35]

**Table 3:** Content of heavy metals in different organs of water dropwort and Chinese National Food Sanitation Standards for heavy metals in vegetables.

Heavy Metals	Root (mg∙kg^−1^)	Stem (mg∙kg^−1^)	Leaf (mg∙kg^−1^)	Standard Limit (mg∙kg^−1^)	Standard Origin
Cu	0.749 ± 0.026 a ^1^	0.032 ± 0.006 c	0.404 ± 0.017 b	10.0	GB15199-94
Cd	0.016 ± 0.001 a	not detected	0.006 ± 0.000 b	0.05	GB15201-94
Pb	0.611 ± 0.001 a	0.054 ± 0.003 b	0.065 ± 0.010 b	0.2	GB14935-94
Cr	5.830 ± 0.031 a	0.102 ± 0.002 c	2.798 ± 0.001 b	0.5	GB14961-94
As	0.809 ± 0.025 a	0.015 ± 0.001 c	0.057 ± 0.001 b	0.5	GB4810-94

^1^ Different letters indicate significant differences between different organs (*p* < 0.05).

**Table 4:** Content of antibiotics and human exposure to antibiotics in water dropwort.

Antibiotics	Aboveground Part (μg∙kg^−1^)	Underground Part (μg∙kg^−1^)	FAO Acceptable Daily Intake (μg) [42]	Human Exposure (μg)
Sulfathiazole	not detected	not detected		
Sulfadimidine	0.1267 ± 0.0088	0.0433 ± 0.0033	2795	0.4928
Sulfamethazine	not detected	not detected		
Ampicillin	not detected	not detected		
Cefalexin	not detected	not detected		
Amoxicillin	not detected	not detected		
Cefotaxime	not detected	not detected		
Penicillin G	not detected	not detected		
Oxytetracycline	0.4067 ± 0.0260	1.0267 ± 0.0727	1677	0.1582
Doxycycline	1.1933 ± 0.0784	3.1133 ± 0.1157	1677	0.4642
Tetracycline	0.9767 ± 0.0869	3.2367 ± 0.1299	1677	0.3799
Aureomycin/Chlortetracycline	1.1833 ± 0.0669	1.2767 ± 0.0606	1677	0.4603
Tilmicosin	not detected	not detected		
Tylosin	not detected	not detected		
Erythromycin	not detected	not detected		
Azithromycin	not detected	not detected		

**Table 5:** Content of different nutrients in different tissues of water dropwort.

Nutrition	Root	Stem	Leaf
Soluble total sugar (mg∙g^−1^)	4.02 ± 0.06 c ^1^	13.61 ± 0.14 a	5.35 ± 0.01 b
Crude protein (mg∙g^−1^)	178.40 ± 1.81 b	159.21 ± 2.17 c	331.46 ± 0.06 a
Crude fat (%)	0.96 ± 0.07 c	1.53 ± 0.09 b	3.38 ± 0.10 a
VC (mg∙100 g^−1^)	1.30 ± 0.02 b	3.90 ± 0.10 a	0.95 ± 0.05 c
Crystalline cellulose (μg∙mg^−1^)	168.15 ± 4.79 b	294.37 ± 1.81 a	112.74 ± 3.26 c
Total lignin (μg∙mg^−1^)	150.56 ± 5.31 a	92.95 ± 2.49 b	140.63 ± 1.58 a
β-VE (μg∙g^−1^)	0.0133 ± 0.00155 a	0.0094 ± 0.00081 b	0.0087 ± 0.00001 b
γ-VE (μg∙g^−1^)	0.1453 ± 0.00551 b	0.026 ± 0.00074 c	1.0693 ± 0.00127 a
α-VE (μg∙g^−1^)	0.6158 ± 0.00692 b	0.2185 ± 0.00488 c	2.2106 ± 0.00377 a
Carotenoids (mg∙100 g^−1^)	0.99 ± 0.02 c	1.94 ± 0.00 b	15.69 ± 0.09 a

^1^ Different letters indicate significant differences between different organs (*p* < 0.05).

## Supplementary Materials

The following are available online at https://www.mdpi.com/article/10.3390/ijerph18168634/s1, Table S1: Sixty-eight different pesticides content in water dropwort used for LW management.

## References

[1] Bai Z., Lee M.R.F., Ma L., Ledgard S., Oenema O., Velthof G.L., Ma W., Guo M., Zhao Z., Wei S. (2018). Global environmental costs of China’s thirst for milk. Glob. Chang. Biol..

[2] Wilkinson J.M., Lee M.R.F. (2018). Review: Use of human-edible animal feeds by ruminant livestock. Animal.

[3] Hu H., Li X., Wu S., Yang C. (2020). Sustainable livestock wastewater treatment via phytoremediation: Current status and future perspectives. Bioresour. Technol..

[4] Hu Y., Cheng H., Tao S. (2017). Environmental and human health challenges of industrial livestock and poultry farming in China and their mitigation. Environ. Int..

[5] Lu Q., He Z.L., Graetz D.A., Stoffella P.J., Yang X. (2010). Phytoremediation to remove nutrients and improve eutrophic stormwaters using water lettuce (*Pistia Stratiotes* L.). Environ. Sci. Pollut. Res..

[6] Ting W.H.T., Tan I.A.W., Salleh S.F., Wahab N.A. (2018). Application of water hyacinth (*Eichhornia crassipes*) for phytoremediation of ammoniacal nitrogen: A review. J. Water Process Eng..

[7] Wang X., Wu X., Yan P., Gao W., Chen Y., Sui P. (2016). Integrated analysis on economic and environmental consequences of livestock husbandry on different scale in China. J. Clean. Prod..

[8] Luo L., He H., Yang C., Wen S., Zeng G., Wu M., Zhou Z., Lou W. (2016). Nutrient removal and lipid production by *Coelastrella* Sp. in anaerobically and aerobically treated swine wastewater. Bioresour. Technol..

[9] Markou G., Wang L., Ye J., Unc A. (2018). Using agro-industrial wastes for the cultivation of microalgae and duckweeds: Contamination risks and biomass safety concerns. Biotechnol. Adv..

[10] Vymazal J. (2011). Constructed wetlands for wastewater treatment: Five decades of experience. Environ. Sci. Technol..

[11] Jabeen R., Ahmad A., Iqbal M. (2009). Phytoremediation of heavy metals: Physiological and molecular mechanisms. Bot. Rev..

[12] Prabakaran K., Li J., Anandkumar A., Leng Z., Zou C.B., Du D. (2019). Managing environmental contamination through phytoremediation by invasive plants: A review. Ecol. Eng..

[13] Doren R.F., Volin J.C., Richards J.H. (2009). Invasive exotic plant indicators for ecosystem restoration: An example from the everglades restoration program. Ecol. Indic..

[14] Xu J., Shen G. (2011). Growing Duckweed in swine wastewater for nutrient recovery and biomass production. Bioresour. Technol..

[15] Cunningham S.D., Ow D.W. (1996). Promises and prospects of phytoremediation. Plant Physiol..

[16] Chen J., Nie Q., Zhang Y., Hu J., Qing L. (2014). Eco-physiological characteristics of *Pistia stratiotes* and its removal of pollutants from livestock wastewater. Water Sci. Technol..

[17] Ekperusi A.O., Sikoki F.D., Nwachukwu E.O. (2019). Application of common duckweed (*Lemna minor*) in phytoremediation of chemicals in the environment: State and future perspective. Chemosphere.

[18] Hu H., Zhou Q., Li X., Lou W., Du C., Teng Q., Zhang D., Liu H., Zhong Y., Yang C. (2019). Phytoremediation of anaerobically digested swine wastewater contaminated by oxytetracycline via *Lemna aequinoctialis*: Nutrient removal, growth characteristics and degradation pathways. Bioresour. Technol..

[19] Xian Q., Hu L., Chen H., Chang Z., Zou H. (2010). Removal of nutrients and veterinary antibiotics from swine wastewater by a constructed macrophyte floating bed system. J. Environ. Manag..

[20] Feng K., Xu Z.-S., Que F., Liu J.-X., Wang F., Xiong A.-S. (2018). An R2R3-MYB transcription factor, *OjMYB1*, functions in anthocyanin biosynthesis in *Oenanthe javanica*. Planta.

[21] Jiang Q., Wang F., Tan H.-W., Li M.-Y., Xu Z.-S., Tan G.-F., Xiong A.-S. (2015). De novo transcriptome assembly, gene annotation, marker development, and miRNA potential target genes validation under abiotic stresses in *Oenanthe javanica*. Mol. Genet. Genom..

[22] Lu C., Li X. (2019). A review of *Oenanthe Javanica* (Blume) DC. as traditional medicinal plant and its therapeutic potential. Evid. Based Complement. Alternat. Med..

[23] Song S., Wang P., Liu Y., Zhao D., Leng X., An S. (2019). Effects of *Oenanthe javanica* on nitrogen removal in free-water surface constructed wetlands under low-temperature conditions. Int. J. Environ. Res. Public Health.

[24] Zhou X., Wang G. (2010). Nutrient concentration variations during *Oenanthe javanica* growth and decay in the ecological floating bed system. J. Environ. Sci..

[25] Chen C., Zhao T., Liu R., Luo L. (2017). Performance of five plant species in removal of nitrogen and phosphorus from an experimental phytoremediation system in the Ningxia irrigation area. Environ. Monit. Assess..

[26] Lu J., Fu Z., Yin Z. (2008). Performance of a water hyacinth (*Eichhornia crassipes*) system in the treatment of wastewater from a duck farm and the effects of using water hyacinth as duck feed. J. Environ. Sci..

[27] Duan M., Zhang Y., Zhou B., Qin Z., Wu J., Wang Q., Yin Y. (2020). Effects of *Bacillus subtilis* on carbon components and microbial functional metabolism during cow manure–straw composting. Bioresour. Technol..

[28] Kim S., Park S.K., Daugherty K.E. (2004). Some physical characteristics and heavy metal analyses of cotton gin waste for potential use as an alternative fuel. Korean J. Chem. Eng..

[29] Malá J., Lagová M. (2014). Comparison of digestion methods for determination of total phosphorus in river sediments. Chem. Pap..

[30] Li J., Xie Z.M., Xu J.M., Sun Y.F. (2006). Risk assessment for safety of soils and vegetables around a lead/zinc mine. Environ. Geochem. Health.

[31] Pan M., Wong C.K.C., Chu L.M. (2014). Distribution of antibiotics in wastewater-irrigated soils and their accumulation in vegetable crops in the pearl river delta, southern China. J. Agric. Food Chem..

[32] Li X., Li Z., Lin C.-J., Bi X., Liu J., Feng X., Zhang H., Chen J., Wu T. (2018). Health risks of heavy metal exposure through vegetable consumption near a large-scale Pb/Zn smelter in central China. Ecotoxicol. Environ. Saf..

[33] Xu C., Wen X., Song W., Zhang Y., Liu H., Wang Y., Qin H., Zhang Z. (2018). Growth responses of *Eichhornia crassipes* to changes of water quality in ecological treatment engineering. Ecol. Environ. Sci..

[34] Tan J., Xu B., Zou F., Jin S., Xu J. (2018). Growth characteristics and nitrogen and phosphorus enrichment ability of *Eichhornia Crassipes* in Wenshanhe village pond water body. J. Green Sci. Technol..

[35] Zhang Y., Zhang Z., Wang Y., Liu H., Wang Z., Yan S., Han Y., Yang L. (2011). Research on the growth characteristics and accumulation ability to N and P of *Eichhornia crassipes* in different water areas of Dianchi Lake. J. Ecol. Rural Environ..

[36] Xia C., Yu D., Wang Z., Xie D. (2014). Stoichiometry patterns of leaf carbon, nitrogen and phosphorous in aquatic macrophytes in eastern China. Ecol. Eng..

[37] Cang L., Wang Y., Zhou D., Dong Y. (2004). Heavy metals pollution in poultry and livestock feeds and manures under intensive farming in Jiangsu Province, China. J. Environ. Sci..

[38] Nicholson F.A., Chambers B.J., Williams J.R., Unwin R.J. (1999). Heavy metal contents of livestock feeds and animal manures in England and Wales. Bioresour. Technol..

[39] Zhang F., Li Y., Yang M., Li W. (2012). Content of heavy metals in animal feeds and manures from farms of different scales in Northeast China. Int. J. Environ. Res. Public Health.

[40] Zhang Q.-Q., Ying G.-G., Pan C.-G., Liu Y.-S., Zhao J.-L. (2015). Comprehensive evaluation of antibiotics emission and fate in the river basins of China: Source analysis, multimedia modeling, and linkage to bacterial resistance. Environ. Sci. Technol..

[41] Schoenmakers K. (2020). How China is getting its farmers to kick their antibiotics habit. Nature.

[42] FAO, WHO (2018). Maximum Residue Limits (MRLs) and Risk Management Recommendations (RMRs) for Residues of Veterinary Drugs in Foods CX/MRL 2-2018. http://www.fao.org/fao-who-codexalimentarius/sh-proxy/en/?lnk=1&url=https%253A%252F%252Fworkspace.fao.org%252Fsites%252Fcodex%252FStandards%252FCXM%2B2%252FMRL2e.pdf.

[43] Yang Y., Wang G., Pan X. (2002). China Food Composition 2002.

[44] Wang S., Lay S., Yu H., Shen S. (2016). Dietary guidelines for chinese residents (2016): Comments and comparisons. J. Zhejiang Univ. Sci. B.

[45] Liu F., Zhang S., Wang Y., Li Y., Xiao R., Li H., He Y., Zhang M., Wang D., Li X. (2016). Nitrogen removal and mass balance in newly-formed *Myriophyllum aquaticum* mesocosm during a single 28-day incubation with swine wastewater treatment. J. Environ. Manag..

[46] Sudiarto S.I.A., Renggaman A., Choi H.L. (2019). Floating aquatic plants for total nitrogen and phosphorus removal from treated swine wastewater and their biomass characteristics. J. Environ. Manag..

[47] Zhou Q., Lin Y., Li X., Yang C., Han Z., Zeng G., Lu L., He S. (2018). Effect of zinc ions on nutrient removal and growth of *Lemna aequinoctialis* from anaerobically digested swine wastewater. Bioresour. Technol..

[48] Lolis L.A., Alves D.C., Fan S., Lv T., Yang L., Li Y., Liu C., Yu D., Thomaz S.M. (2020). Negative correlations between native macrophyte diversity and water hyacinth abundance are stronger in its introduced than in its native range. Divers. Distrib..

[49] Wang H., Wang Q., Bowler P., Xiong W. (2016). Invasive aquatic plants in China. Aquat. Invasions.

[50] Zheng Z.C., Li T.X., Zeng F.F., Zhang X.Z., Yu H.Y., Wang Y.D., Liu T. (2013). Accumulation characteristics of and removal of nitrogen and phosphorus from livestock wastewater by *Polygonum hydropiper*. Agric. Water Manag..

[51] Akansha J., Nidheesh P.V., Gopinath A., Anupama K.V., Suresh Kumar M. (2020). Treatment of dairy industry wastewater by combined aerated electrocoagulation and phytoremediation process. Chemosphere.

[52] Chang J., Wu S., Dai Y., Liang W., Wu Z. (2013). Nitrogen removal from nitrate-laden wastewater by integrated vertical-flow constructed wetland systems. Ecol. Eng..

[53] Mayo A.W., Hanai E.E., Kibazohi O. (2014). Nitrification–denitrification in a coupled high rate—Water hyacinth ponds. Phys. Chem. Earth Parts A B C.

[54] Sasse J., Martinoia E., Northen T. (2018). Feed your friends: Do plant exudates shape the root microbiome?. Trends Plant Sci..

[55] Shen Y., Ji Y., Li C., Luo P., Wang W., Zhang Y., Nover D. (2018). Effects of phytoremediation treatment on bacterial community structure and diversity in different petroleum-contaminated soils. Int. J. Environ. Res. Public Health.

[56] Abe K., Komada M., Ookuma A., Itahashi S., Banzai K. (2014). Purification performance of a shallow free-water-surface constructed wetland receiving secondary effluent for about 5 years. Ecol. Eng..

[57] Duan J., Feng Y., Yu Y., He S., Xue L., Yang L. (2016). Differences in the treatment efficiency of a cold-resistant floating bed plant receiving two types of low-pollution wastewater. Environ. Monit. Assess..

[58] Wilkie A.C., Evans J.M. (2010). Aquatic plants: An opportunity feedstock in the age of bioenergy. Biofuels.

[59] Xu J., Cui W., Cheng J.J., Stomp A.-M. (2011). Production of high-starch duckweed and its conversion to bioethanol. Biosyst. Eng..

[60] Cui W., Cheng J.J. (2015). Growing duckweed for biofuel production: A review. Plant Biol..

[61] Abubackar H.N., Keskin T., Arslan K., Vural C., Aksu D., Yavuzyılmaz D.K., Ozdemir G., Azbar N. (2019). Effects of size and autoclavation of fruit and vegetable wastes on biohydrogen production by dark dry anaerobic fermentation under mesophilic condition. Int. J. Hydrogen Energy.

[62] Dismukes G.C., Carrieri D., Bennette N., Ananyev G.M., Posewitz M.C. (2008). Aquatic phototrophs: Efficient alternatives to land-based crops for biofuels. Curr. Opin. Biotechnol..

