# Supplementary material for: A New Green Model for the Bioremediation and Resource Utilization of Livestock Wastewater
Source: Int J Environ Res Public Health. 2021 Aug 16;18(16):8634. doi: 10.3390/ijerph18168634 (PMC8391518; doi:10.3390/ijerph18168634)
Supplement: Supplementary file 1 [file ijerph-18-08634-s001.zip › ijerph-1317496-supplementary.pdf]

Table S1 Sixty-eight different pesticides content in water dropwort used for LW management

No.	Item	Unit	Standard	Content	Detection threshold	Single conclusion	Test method
1	Methamidophos	$mg{\cdot}kg^{-1}$	≤ 0.05	not detected	0.01	Meet standard	NY/T 761-2008 Method 2, Part 1
2	Parathion	$mg{\cdot}kg^{-1}$	≤ 0.01	not detected	0.008	Meet standard	GB/T 5009.145-2003
3	Methyl parathion	$mg{\cdot}kg^{-1}$	≤ 0.02	not detected	0.02	Meet standard	NY/T 761-2008 Method 2, Part 1
4	Hexachlorocyclohexane	$mg{\cdot}kg^{-1}$	≤ 0.05	not detected	0.0004	Meet standard	NY/T 761-2008 Method 2, Part 2
5	Phorate	$mg{\cdot}kg^{-1}$	≤ 0.01	not detected	0.01	Meet standard	GB 23200.8-2016
6	Omethoate	$mg{\cdot}kg^{-1}$	≤ 0.02	not detected	0.02	Meet standard	NY/T 761-2008 Method 2, Part 1
7	Isocarbophos	$mg{\cdot}kg^{-1}$	≤ 0.05	not detected	0.03	Meet standard	NY/T 761-2008 Method 2, Part 1
8	Sofenphos-methyl	$mg{\cdot}kg^{-1}$	≤ 0.01	not detected	0.004	Meet standard	GB/T 2009.144-2003
9	Carbofuran	$mg{\cdot}kg^{-1}$	≤ 0.02	not detected	0.01	Meet standard	NY/T 761-2008 Part 3
10	Aldicarb	$mg{\cdot}kg^{-1}$	≤ 0.03	not detected	0.009	Meet standard	NY/T 761-2008 Part 3
11	Chlorpyrifos	$mg{\cdot}kg^{-1}$	/	not detected	0.02	/	NY/T 761-2008 Method 2, Part 1
12	Triazophos	$mg{\cdot}kg^{-1}$	/	not detected	0.01	/	NY/T 761-2008 Method 2, Part 1
13	Dimethoate	$mg{\cdot}kg^{-1}$	/	not detected	0.02	/	NY/T 761-2008 Method 2, Part 1
14	Acephate	$mg{\cdot}kg^{-1}$	≤ 1	not detected	0.03	Meet standard	NY/T 761-2008 Method 2, Part 1
15	Lannate	$mg{\cdot}kg^{-1}$	≤ 0.2	not detected	0.01	Meet standard	NY/T 761-2008 Part 3
16	Fenvalerate and S-fenvalerate (Measured as Fenvalerate)	$mg{\cdot}kg^{-1}$	/	not detected	0.002	/	NY/T 761-2008 Method 2, Part 2
17	DDVP	$mg{\cdot}kg^{-1}$	≤ 0.2	not detected	0.01	Meet standard	NY/T 761-2008 Method 2, Part 1
18	Profenofos	$mg{\cdot}kg^{-1}$	/	not detected	0.04	/	NY/T 761-2008 Method 2, Part 1
19	Fenitrothion	$mg{\cdot}kg^{-1}$	≤ 0.5	not detected	0.02	Meet standard	NY/T 761-2008 Method 2, Part 1
20	Diazinon	$mg{\cdot}kg^{-1}$	/	not detected	0.02	/	NY/T 761-2008 Method 2, Part 1
21	Malathion	$mg{\cdot}kg^{-1}$	/	not detected	0.03	/	NY/T 761-2008 Method 2, Part 1
22	Phosemet	$mg{\cdot}kg^{-1}$	/	not detected	0.06	/	NY/T 761-2008 Method 2, Part 1
23	Phosalone	$mg{\cdot}kg^{-1}$	/	not detected	0.05	/	NY/T 761-2008 Method 2, Part 1
24	Phoxim	$mg{\cdot}kg^{-1}$	≤ 0.05	not detected	0.01	Meet standard	GB/T 5009.102-2003
25	Cypermethrin and Efficient cypermethrin	$mg{\cdot}kg^{-1}$	/	not detected	0.003	/	NY/T 761-2008 Method 2, Part 2

	(measured as cypermethrin)						
26	Fenpropathrin	$mg \cdot kg^{-1}$	/	not detected	0.002	/	NY/T 761-2008 Method 2, Part 2
27	Cyhalothrin and Efficient cyhalothrin (measured as cyhalothrin)	$mg \cdot kg^{-1}$	/	not detected	0.0005	/	NY/T 761-2008 Method 2, Part 2
28	Cyfluthrin and Efficient cyfluthrin (measured as cyfluthrin)	$mg \cdot kg^{-1}$	/	not detected	0.002	/	NY/T 761-2008 Method 2, Part 2
29	Deltamethrin	$mg \cdot kg^{-1}$	/	not detected	0.001	/	NY/T 761-2008 Method 2, Part 2
30	Bifenthrin	$mg \cdot kg^{-1}$	/	not detected	0.0006	/	NY/T 761-2008 Method 2, Part 2
31	Fluvalinate	$mg \cdot kg^{-1}$	/	not detected	0.002	/	NY/T 761-2008 Method 2, Part 2
32	Flucythrinate	$mg \cdot kg^{-1}$	/	not detected	0.001	/	NY/T 761-2008 Method 2, Part 2
33	Triadimefon	$mg \cdot kg^{-1}$	/	not detected	0.025	/	GB/T 23200.8-2016
34	Chlorothalonil	$mg \cdot kg^{-1}$	/	not detected	0.0003	/	NY/T 761-2008 Method 2, Part 2
35	Iprodione	$mg \cdot kg^{-1}$	/	not detected	0.001	/	NY/T 761-2008 Method 2, Part 2
36	Carbaryl	$mg \cdot kg^{-1}$	≤ 1	not detected	0.01	Meet standard	NY/T 761-2008 Part 3
37	Dicofol	$mg \cdot kg^{-1}$	/	not detected	0.0008	/	NY/T 761-2008 Method 2, Part 2
38	Procymidone	$mg \cdot kg^{-1}$	/	not detected	0.002	/	NY/T 761-2008 Method 2, Part 2
39	Pentachloronitrobenzene	$mg \cdot kg^{-1}$	/	not detected	0.00027	/	GB/T 5009.19-2008 Method 1
40	Vinclozolin	$mg \cdot kg^{-1}$	/	not detected	0.0001	/	NY/T 761-2008 Method 2, Part 2
41	Carbendazim	$mg \cdot kg^{-1}$	/	not detected	0.00012	/	GB/T 20769-2008
42	Imidacloprid	$mg \cdot kg^{-1}$	/	not detected	0.0055	/	GB/T 20769-2008
43	Fipronil	$mg \cdot kg^{-1}$	≤ 0.02	not detected	0.002	Meet standard	SN/T 1982-2007
44	Acetamiprid	$mg \cdot kg^{-1}$	/	not detected	0.01	/	GB/T 23584-2009
45	Pyridaben	$mg \cdot kg^{-1}$	/	not detected	0.00304	/	GB/T 20769-2008
46	Difenoconazole	$mg \cdot kg^{-1}$	/	not detected	0.075	/	GB 23200.8-2016
47	Pyrimethanil	$mg \cdot kg^{-1}$	/	not detected	0.00017	/	GB/T 20769-2008
48	Emamectin Benzoate	$mg \cdot kg^{-1}$	/	not detected	0.00008	/	GB/T 20769-2008
49	Dimethomorph	$mg \cdot kg^{-1}$	/	not detected	0.00018	/	GB/T 20769-2008
50	Chlorfenapyr	$mg \cdot kg^{-1}$	/	not detected	0.025	/	GB 23200.8-2016
51	Prochloraz and Prochloraz-manganese chloride complex	$mg \cdot kg^{-1}$	/	not detected	0.005	/	NY/T 1456-2007
52	Azoxystrobin	$mg \cdot kg^{-1}$	/	not detected	0.01	/	NY/T 1456-2007
53	Pendimethalin	$mg \cdot kg^{-1}$	/	not detected	0.05	/	GB 23200.8-2016

54	Thiamethoxam	$mg\cdot kg^{-1}$	/	not detected	0.05	/	GB 23200.8-2016
55	Chlorfluazuron	$mg\cdot kg^{-1}$	/	not detected	0.0376	/	GB 23200.8-2016
56	Chlorobenzuron	$mg\cdot kg^{-1}$	/	not detected	0.03	/	GB/T 5009.135-2003
57	Cyromazine	$mg\cdot kg^{-1}$	/	not detected	0.02	/	NY/T 1725-2009
58	Metalaxyl and metalaxyl-M (Measured as metalaxyl)	$mg\cdot kg^{-1}$	/	not detected	0.0376	/	GB 23200.8-2016
59	Propamocarb and propamocarb hydrochloride (Measured as propamocarb)	$mg\cdot kg^{-1}$	/	not detected	0.00002	/	GB/T 20769-2008
60	Paclobutrazol	$mg\cdot kg^{-1}$	/	not detected	0.00014	/	GB/T 20769-2008
61	Forchlorfenuron	$mg\cdot kg^{-1}$	/	not detected	0.0057	/	GB/T 20770-2008
62	Chlorantraniliprole	$mg\cdot kg^{-1}$	/	not detected	/	/	GB/T 20769-2008
63	Permethrin	$mg\cdot kg^{-1}$	≤ 1	not detected	0.001	Meet standard	NY/T 761-2008 Method 2, Part 2
64	Etofenprox	$mg\cdot kg^{-1}$	/	not detected	0.01	/	SN/T 2151-2008
65	Tebufenozide	$mg\cdot kg^{-1}$	/	not detected	0.00695	/	GB/T 20769-2008
66	Pyraclostrobin	$mg\cdot kg^{-1}$	/	not detected	0.00013	/	GB/T 20769-2008
67	Avermectin	$mg\cdot kg^{-1}$	/	not detected	0.01	/	GB 23200.19-2016
68	Diflubenzuron	$mg\cdot kg^{-1}$	/	not detected	0.04	/	GB/T 5009.147-2003
